# Enhancing e-commerce recommendation systems through approach of buyer's self-construal: necessity, theoretical ground, synthesis of a six-step model, and research agenda

**DOI:** 10.3389/frai.2023.1167735

**Published:** 2023-05-24

**Authors:** Yilin Feng

**Affiliations:** Institute for Digital Technologies, Loughborough University London, London, United Kingdom

**Keywords:** self-construal, collaborative filtering algorithm, grounded theory (GT), psychology informed, cold-start problem, data-sparsity problem

## Abstract

The current recommendation system predominantly relies on evidential factors such as behavioral outcomes and purchasing history. However, limited research has been conducted to explore the use of psychological data in these algorithms, such as consumers' self-perceived identities. Based on the gap identified and the soaring significance of levering the non-purchasing data, this study presents a methodology to quantify consumers' self-identities to help examine the relationship between these psychological cues and decision-making in an e-commerce context, focusing on the projective self, which has been overlooked in previous research. This research is expected to contribute to a better understanding of the cause of inconsistency in similar studies and provide a basis for further exploration of the impact of self-concepts on consumer behavior. The coding method in grounded theory, in conjunction with the synthesis of literature analysis, was employed to generate the final approach and solution in this study as they provide a robust and rigorous basis for the findings and recommendations presented in this study.

## 1. Introduction

Individuals have access to a plethora of information in the modern digital age. However, the difficulty is in efficiently targeting the desired audience and achieving a meaningful advertising impact. In this context, recommendation algorithms and search engines significantly facilitate individuals' access to the desired information (Ries and Trout, 2001). For example, the development of ChatGPT, a generative pre-trained transformer (GPT), exemplifies the evolution of recommendation systems. Instead of delivering original content, the program concentrates on processing enormous quantities of raw data and offering personalized information to users. ChatGPT is viewed as a formidable threat to firms such as Google due to its capabilities and is referred to as “the most disruptive technology” by Haque et al. (2022). Likewise, the e-commerce industry must adjust to these shifting conditions. Rather than depending on consumers to navigate and find what they are looking for, e-commerce websites must seriously consider their customers' needs, wants, and preferences. This could be accomplished by utilizing a system that blends “consumers” psychological cues into the algorithm for making recommendations. The outcome would be the provision of more precise and individualized recommendations, saving consumers time and effort (Lex et al., 2021).

The main challenge is to improve those algorithms. Computer scientists have created powerful and complex mathematical algorithms that derive relatively accurate predictions from behavioral data such as reviews, user profiles, and browsing data. However, two prominent drawbacks exist as barriers that prevent a more robust application of artificial intelligence for marketing practices. First, the process of those conclusions is invisible and impossible for marketers to track it under the current user data protection system. This creates two significant problems: the cold-start problem and the data-sparsity problem. Cold-start happens when consumers first interact with the algorithm, and inaccurate results are provided; Data sparsity problem sometimes happens when there are many products and consumers (Zhang et al., 2021). Furthermore, their lack of explainability makes the recommendation systems less able to self-correct and transfer to other scenarios. Second, data input for user response is mainly reckoned as behavioral and becomes an oversimplification while more hidden customer mentality is not seen and used for analysis. This oversimplification of using only behavioral data is distanced from the precise prediction of consumer decisions embedded in a complex socioeconomic system with multi-facet features of psychological and social influences. From the perspective of method development, furthermore, there calls for a non-platform-based data field for experimenting, extracting, reasoning, and testing data and work as a catalyst for the feasibility and viability of using a new approach to generate the best items that rest well on consumer's unspoken mindsets.

In this sense, the user's psychology might be a key hurdle for the breakthrough in analyzing consumer behaviors. The development of recommendation systems requires human psychology, such as emotions, intuitions, and desires, which is studied by social scientists and biologists, and the implementation of logic and mathematics to recognize and analyze the mass amount of unstructured data (or so-called big data) to provide insights (Harari, 2017). However, a recent survey by Lex suggests that most algorithms nowadays are still driven by consumers' behavioral data, which means the study of recommendation systems is not balanced. Such algorithms developed nowadays have not been taught how to interpret human behaviors on a deeper level because the underlying psychological frameworks were not taught to them (Lex et al., 2021).

The present investigation is organized methodically and systematically. We begin with the investigation's justification, emphasizing the study's significance in the research field and its relevance to current practices. The section on methodology describes the evolution of the research approach and the stages that led to the recent investigation. The results section provides an exhaustive structure. Finally, the study's limitations and prospective future research pathways are reviewed, highlighting the areas needing further exploration and development.

## 2. Background and related work

This section comprehensively reviewed the literature on algorithms and construal-self theory in the e-commerce context. Our research focuses on the actual-self, ideal-self, brand-self, influencer-self, and projective-self self-image. It uses a constructivist grounded theory technique for brand-self and influencer-self. This section illustrates the initial phase of the literature study, which serves to define our research question, enhance the specificity of the coding process for brand-self and influencer-self, and provide insight into the evolution of the idea of projective-self. The literature review is then utilized to get a greater grasp of actual-self and ideal-self, which serve as the basis of our investigation.

### 2.1. Current recommendation systems

Content filtering and collaborative filtering are the two primary classifications that may be applied to the existing recommendation systems. The most prevalent method is collaborative filtering, and many systems opt for a hybrid method that combines content filtering and collaborative filtering to enhance benefits. Utilizing consumer activities such as reviews and ratings, collaborative filtering approaches combine users with similar characteristics (referred to as a “neighborhood”). Then, recommendations are generated based on the products chosen or rated by people in the same neighborhood. In contrast, content filtering algorithms provide suggestions based on the attributes of goods that a user prefers, such as price, color, and material.

Despite the growing popularity of complex algorithms such as neural networks in recent years, collaborative filtering continues to be the method of choice for recommendation systems for several reasons. First, the algorithm must produce results in a timely manner, as neural networks frequently require extensive training time to create accurate predictions. Second, training neural networks requires an enormous quantity of data, which is not always available. Finally, explainability is a crucial component of recommendation systems. However neural networks may not give the requisite amount of transparency (Sharma and Gera, 2013; Liao and Sundar, 2021). Despite the limitations of neural networks, some academic studies suggest that incorporating neural networks with collaborative filtering can lead to improved results (Wei et al., 2017).

Given these considerations, the hybrid approach of combining content filtering and collaborative filtering offers a balance between accuracy, speed, and explainability. The neighborhood-based approach provides a clear and concise explanation for recommendations while also delivering results in real-time. By incorporating elements of content filtering, the hybrid approach can further enhance the overall performance of the recommendation system (Kumar and Thakur, 2018).

The field of recommendation systems has undergone extensive research with a diverse range of focuses. Some studies have concentrated on optimizing price and profit, whereas others have centered on enhancing member retention and mid-term engagement (Gomez-Uribe and Hunt, 2015; Jannach and Adomavicius, 2017). In our research, we assess the efficacy of recommendation systems using the REAN framework, which encompasses reach, engagement, activation, and nurture (Jackson, 2009).

### 2.2. Construal-self theories

The self-construal theory of self-concepts is selected as the conceptual framework for developing a comprehensive model of self-concepts for the use case of e-commerce websites in this research. This is due to its significance in portraying who we are as individuals since our pets, positions, and online behavior reveal a great deal about us in the modern digital world.

The self-construal theory, which academics have extensively examined in a variety of domains for over four decades, offers numerous dimensions and is carefully chosen for this study. The five dimensions traced in this study are the actual-self, the ideal self, the projective self, the brand-self, and the influencer-self. However, the self-concepts hypothesis has been criticized, with one relevant argument being the extended-self theory. According to this theory, consumers' attachment to positions is a component of their self-identity, with brand-self-identity, influencer-self-identity, and projective self-identity being external self-identities. Consumers expand their sense of self to external objects, such as brands and influencers. Cohen raises concerns about the hazy boundaries of the extended self, although Belk later clarifies that the extended self is distinct from one's key possessions (since one may be sentimentally attached to an item but not necessarily consider it to be their self-identity), providing clarity on the subject (Cohen, 1989; Belk, 2013).

Scholars have intensively researched the actual-self and ideal self for decades. Consequently, the metrics for these two notions were created through a synthesis of literary analysis. The identity of actual-self, also known as the “true self”, “fundamental self”, or simply “self”, is the perception of how one perceives oneself and how he interprets the actions of others (Higgins, 1996). Those traits by which one understands himself are considered steady properties. The actual-social self, on the other hand, is defined as “how people believe they are seen by significant others” (Hakimian et al., 2014) and is characterized by prevention-focused (non-losses) behavior that ensures safety and security (Higgins, 1996, 1997; Toyama, 2022). The ideal self is sometimes referred to as the “desired self” or “idealized image” and is described as how people would like to perceive themselves (Sirgy, 1982; Hong and Zinkhan, 1995; Hakimian et al., 2014). In the consumption process, consumers seek to integrate their desired traits into their ideal self (Mathews, 2015; Dib and Johnson, 2019) and have more variety-seeking behaviors to satisfy their need for intense stimulation (Menon and Kahn, 1995).

The concepts of brand-self and influencer-self were established through a grounded theoretical coding approach due to the limited research in the e-commerce context and the abundance of available data from online sources. Brand-self, which could also be referred to as brand-extended self-construal (David and Sandor, 2006), is defined as individuals seeing themselves through the self-brand connection channeled by brand anthropomorphism (Belk, 2014b). Simply put, brand-self is the identity people adopt while projecting themselves onto a brand (Belk, 2014a,b; Matzler et al., 2016). The formation of brand-self for consumers occurs through interaction with the brand, as brands use mascots, brand attributes, and brand spokespersons to form brand personality and create an emotional attachment with (Kwon and Mattila, 2015). A brand is defined as “a name, term, sign, symbol, or design, or combination of them which is intended to identify the goods and services of one seller or group of sellers and to differentiate them from those of competitors” (Keller, 1993; Kotler, 1994), requiring mediations such as products and services to do so. Brands can construct and present consumers' self-identity, with consumers' personalities potentially being defined by their attitudes toward the products they use and the image those products present (Fisk and Tucker, 1968). Influencer-self is the self-image we possess when we evaluate an influencer, and it is our projection of influencers. It is considered an influencer-extended self-concept and can be positive or negative depending on our connection with different influencers. Influencers are individuals with expertise in a specific category or product who are influential in changing' people's opinions, behaviors, and attitudes due to their halo effect (Maragheh et al., 2018). To maintain their status as influencers, they must consistently create high-quality content in various forms, such as opinions, videos, advice, or photos. The process of becoming an influencer involves internalization, identification, and compliance according to Kelman's receiver processing modes (Kelman, 2017; Jun and Yi, 2020).

Our research sheds light on the crucial significance of the projective self in consumer behavior. Despite previous studies that have surveyed consumers on their perceptions of actual-self, ideal-self, and certain brands, to understand the impact of brand-self congruence on purchasing choices, these studies have neglected the contextual factors that influence consumer decision-making. In particular, the event for which a product is needed, and the intended use of the product have been overlooked as crucial aspects that must be considered. The concept of the projective self was derived from the author's literature review of the other four self-concepts inspired by Meng's research, which revealed a gap in the existing theory (Meng and Wang, 2019). Most studies in this field fail to identify the context in which the four self-concepts are framed or the process through which this occurs. For instance, the absence of consistency in considering the situational context in which consumers answer questions about their purchasing intentions and the impact of the variety of these circumstances on the measurement of different self-concepts remains an undiscussed issue in the existing literature (Hughes, 1971; Hermanda et al., 2019). Expanding our understanding of projective self, this research seeks to bridge this gap and provide a more comprehensive and nuanced understanding of consumer behavior.

Our research has shown ten gaps between the five self-concepts that occur in consumer behavior. By understanding the links between these gaps, recommendation systems may optimize the personalization of recommendations for specific customers by making educated and adaptable judgments. The ability to exploit these gaps enables a more dynamic approach to personalization, allowing the recommendation engine to make rapid and flexible decisions. This can raise the effectiveness of the recommendation system and improve the consumer experience. By thoroughly comprehending the interplay between these self-concepts and the gaps between them, the recommendation system can generate data-driven judgments that are personalized to the individual needs and preferences of each consumer, resulting in more satisfying suggestions.

## 3. Gaps

### 3.1. Theoretical gaps

Despite extensive research on the relationship between consumers' actual-self and ideal-self in e-commerce settings, there remains a noticeable gap in developing a comprehensive theory on the construal self in these contexts (Belk, 2013). It was believed that consumers fulfilled their aspiration of finding a settled-down self-identity as a state of ideal self-identity through choosing their satisfactory items. While much has been done investigating the roles of brands and influencers in guiding and influencing consumers' behaviors, the significance of both brands and influencers as part of consumers' self-identity has received limited attention in the academic literature (Sharon Wu et al., 2015), possibly due to inability of finding the suitable measures without awakening consumer' awareness. To comprehend their impact on consumer decision-making, it is necessary to further conceptualize these notions within the context of construal-self in e-commerce settings. This theoretical gap demonstrates the need for additional research in this area and presents potential for future studies to establish a thorough grasp of the construal self in e-commerce scenarios.

### 3.2. Methodological gaps

A thorough review of the strategies used to develop an optimal recommendation system algorithm reveals a variety of approaches. While computer scientists primarily concentrate on developing models from the technical perspective so that the models can predict users' behaviors more accurately and efficiently rather than understanding the psychological cues underlying those behaviors, while social scientists focus on “consumers” psychological states. However, an absence of studies has effectively integrated psychological cues experienced by consumers into the algorithms. Our research bridges the gap between these two domains by proposing a set of quantifiable codes that can be seamlessly integrated into an algorithm (Lex et al., 2021). Additionally, the data required for these codes are readily available in multiple forms, making them easily accessible for implementation. The methodology employed in this study is advanced in its ability to directly measure metrics during the consumer purchasing experience, as opposed to many traditional social research methods such as interviews and surveys. These traditional methods often require a substantial amount of time to analyze and can lack context regarding the actual purchasing process. Furthermore, researchers can acquire valuable insights into consumer behavior and preferences in real-time and in a setting directly relevant to the purchasing experience by employing the proposed methodology.

### 3.3. Practical gaps

A plethora of studies in E-commerce has proposed solutions that fall short in terms of feasibility and practicality for industry implementation. This can be attributed to various reasons, such as the unavailability of technology or the rigorous requirements made by their methodology. However, our proposed solution has been meticulously evaluated for its feasibility, validity, and applicability to ensure that it is ready for immediate implementation in the industry. This meticulous examination distinguishes our solution from prior research on the subject and makes it a likely candidate for adoption by the industry.

## 4. Method

This qualitative investigation is studied through the lens of interpretivism and employs the grounded theory and synthesis of literary analysis. The interpretivist study is a typical research philosophy that emphasizes the accumulation of people's subjective experiences and perspectives as opposed to a singular fact. It emphasizes how social institutions and other environmental factors influence human behavior. The grounded theory technique informed by constructivism and the interpretivist paradigm permits a nuanced and comprehensive understanding of the research topic.

The synthesis of a literary analysis study serves two purposes: first, it directs the research topics. Second, it aids in formulating the ultimate theory. Synthesis of literary analysis is the methodology that systematically synthesizes previous research. Compared to the literature review, which critiques previous studies, it focuses more on generating a comprehensive overview of the existing knowledge and theories. From the large realm of marketing research, the synthesis of literary analysis assists in narrowing the emphasis and formulating more precise research questions. The actual-self and the ideal self are well-established ideas being investigated by past researchers. There are some studies on branded-self and influencer-self. Therefore, we formulate a generic inquiry to explore these two aspects. Finally, the projective-self is inducted by the author inspired by Meng's research (Meng and Wang, 2019). The final theory is developed by synthesizing all five concepts. This process helps to make the final theory more reliable and with more dimensions. One of the problems with only using the grounded theory is that the final theory could be too simple and unreliable.

The core idea of grounded theory is that we are only supposed to have a general research question and let the code emerge itself. One of the significant differences between those theories is how much literature research has been conducted (Qureshi and Ünlü, 2020). The classic ground theory school attempts to free researchers completely from the literature, so the literature is done after the data analysis; the interpretivist ground theory school allows researchers to have prior knowledge to increase the sensitivity of the coding process; and the constructivist challenges researchers to use their prior knowledge critically and effectively. They are required to be aware of and constantly examine how their prior knowledge is impacting their coding process. The literature review (synthesis of literal analysis) should be done as researchers find it appropriate to help researchers code with critical thinking. That knowledge should be compared at the end of the coding process. The advantage of constructivist grounded theory is that it is both practical, as it is developed with a purpose, and also that it considers the perspectives of the participants (in this sense, the consumers), and it offers more flexibility as a researcher to interpret the research topic. It helps researchers to be specific in their study. The approach focuses on accumulating knowledge through people's experiences and subjective opinions. The research questions are encouraged to be altered based on the collected data. It encourages the researchers to first code all the pieces of data. Also, it frees researchers to use their personal experiences and tacit knowledge to draw conclusions that are usually unobservable. They encourage researchers to use literary analysis synthesis at any research stage to help them develop practical and comprehensive theoretical codes (Sebastian, 2019).

“Research question: What are the factors of ideal-self, actual-self, brand-self, influencer-self, and projective-self that influence consumer behaviors in e-commerce settings?”

**Figure 1:**
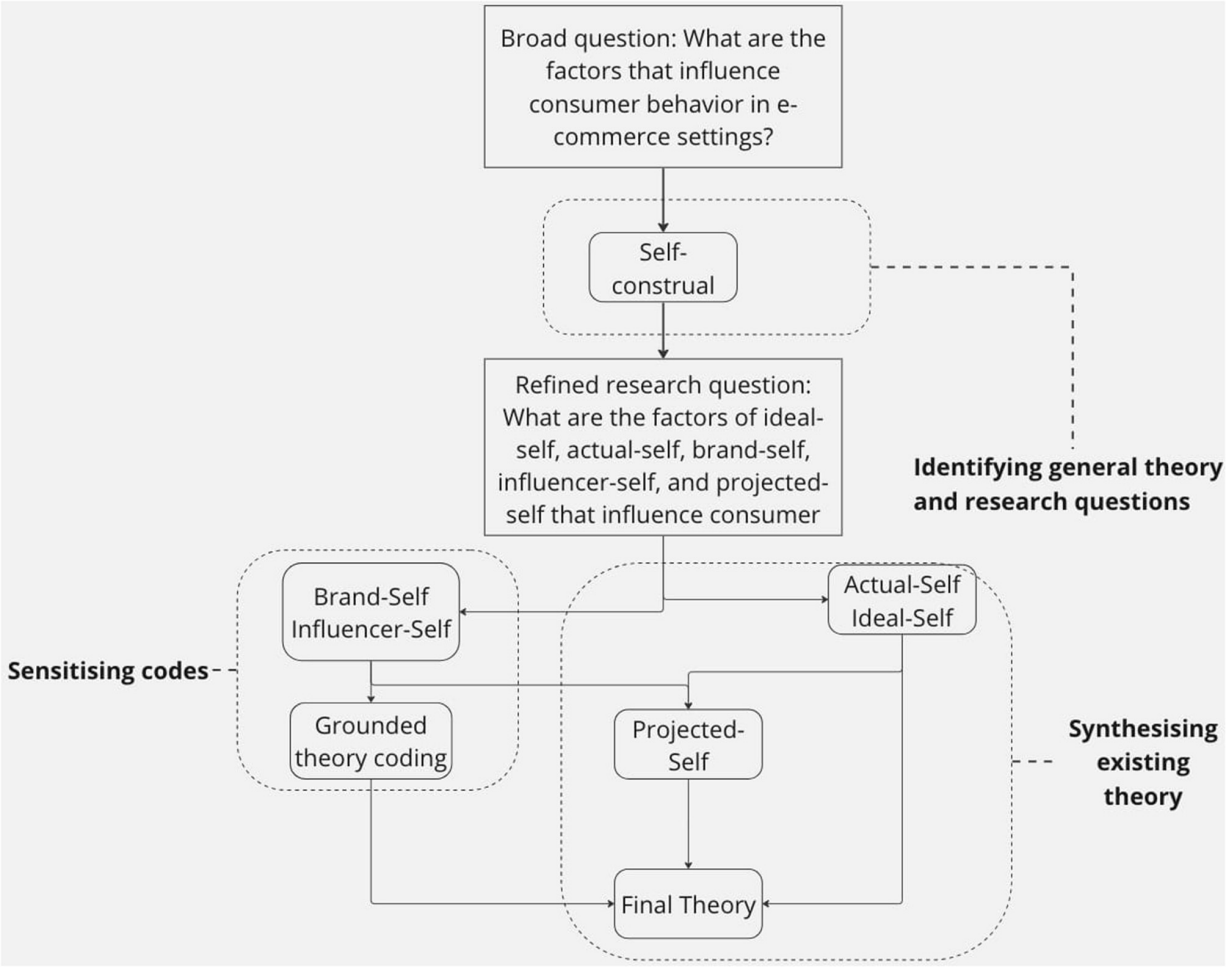
Diagram of theory checking and code sensitizing procedure.

We, therefore, regard synthesis of cross-disciplinary literary analysis as a tool in our research for the following two reasons: The empirical studies that have been operated in the past offer us abundant and reliable data sources to scope out our research questions and to generate the theoretical framework; it facilitates in the coding process to gear our codes toward higher sensitivity to the research projective (Oyibo et al., 2022). The literature review is used, as shown in Figure 1. First, this research refers to a large body of studies on consumers' self-construal theory, the synthesis of literary analysis helps narrow down the research question. Second, it spots a few plausibly interconnected concepts during the coding process, and the synthesis of literary analysis helps sensitize the codes; Last, it gives a guideline in the final step of reconstructing the theory.

The combination of grounded theory's coding approach and literate analysis's synthesis allows for an in-depth understanding of the topic. The out method is chosen to tailor the structure and size of the data we fetch. We do not code bibliometrics using grounded theory like the method Walsh proposed suggests—BIBGT (BIB = Bibliometrics; GT = Grounded theory), although our methods do have many similarities such as our sampling techniques and synthesizing techniques (Walsh and Rowe, 2022). The method of synthesis of literary analysis was created by Erik Mueggler to synthesize a large amount of literature from various disciplines (Mueggler, 2001). It is used in three ways in our research: (1) identifying the general theory and research questions, (2) synthesizing existing theories on ideal-self and actual-self in e-commerce settings, and (3) synthesizing all five self-identities (ideal-self, actual-self, brand-self, influencer-self, and projective-self) to arrive at the final theory. The construal-self theory is chosen as the overarching framework to understand multi-dimensional consumer experiences. The literature review is crucial in identifying gaps in the field and synthesizing existing theories and knowledge. Abundant online data can be used to answer questions about the impact of brands and influencers on consumer behavior through the use of grounded theory's coding method. Furthermore, the researcher's tacit knowledge is necessary to extract the underlying information in the online data.

### 4.1. Data collection

Charmaz emphasizes the practical application of grounded theory in research by utilizing various types of data: social media resources, blogs, and empirical data websites. This approach allows for a comprehensive exploration of the limitations and benefits of each data source (Brink et al., 2006). For example, YouGov offers information such as their survey results of the brand's popularity level. Still, they do not provide information such as the style of the brand or the personality of the brand. While in blogs such as makeup savvy, you would see text like “luxurious silky cleanser suitable for all skin types”, in this sentence, three codes could be delivered: the functionality of the product, the target audience of the product and the brand prestige. However, the researcher believes the word luxury does not describe the brand prestige for the mass, but rather how the user felt while using this product. Therefore, this code was not considered necessary in the initial sampling. In other words, social media resources provide insight into the branding of products through images and customer comments; Blogs offer information about brand prestige and history, as well as influencer ethnicity and experiences; Online survey research websites, such as YouGov, provide sales statistics and popularity data for brands and influencers (Twyman, 2008). The goal is to maximize the use of data and tools to answer research questions effectively.

The technique of constructive grounded theory emphasizes code reflection and the concurrent production of new codes and theories (Sebastian, 2019). To obtain useful data, data collection and analysis should occur simultaneously. Beginning with purposeful sampling, data are gathered based on their relevance to research questions. After accumulating knowledge of the codes, a preliminary theory can be formulated. The gaps in the theory are then filled via theoretical sampling to acquire data pertaining to the theoretical framework (Qureshi and Ünlü, 2020).

### 4.2. Data analysis

#### 4.2.1. Web-based data analysis

The data collection process using online forums, questionnaires, blogs, statistics, and social media is commonly referred to as “web-based data collection”. This method is becoming increasingly popular in marketing and sociology research. It is beneficial for complementing other research methodologies when data is insufficient to comprehensively understand the topic. To minimize bias, researchers must be careful to ensure the reliability and validity of the collected data. The benefits of collecting data from online resources include access to a global audience, individual and group consumer data, already-formatted text and images for easier coding, and the ability to gather diverse data types, such as surveys (Griffiths, 2010).

#### 4.2.2. Charmaz's method of coding

Adopting Charmaz's approach to coding, we begin by developing codes, categorizing them and finally generalizing them into themes. Charmaz emphasizes the importance of initially developing a multitude of codes and selecting the most relevant codes for the study based on their importance and frequency. Keeping detailed memos throughout the process is crucial for building a robust theory based on the codes (Charmaz, 2014). The process then repeats, where we reflect on the codes to gather relevant data and form new codes. This approach allows the researcher to utilize their experiences and tacit knowledge, especially in research areas where a significant amount of information is unobservable. Additionally, the iterative nature of the coding process allows for continuous refinement and improvement of the theory (Kathy, 2006).

### 4.3. Procedure

**Figure 2:**
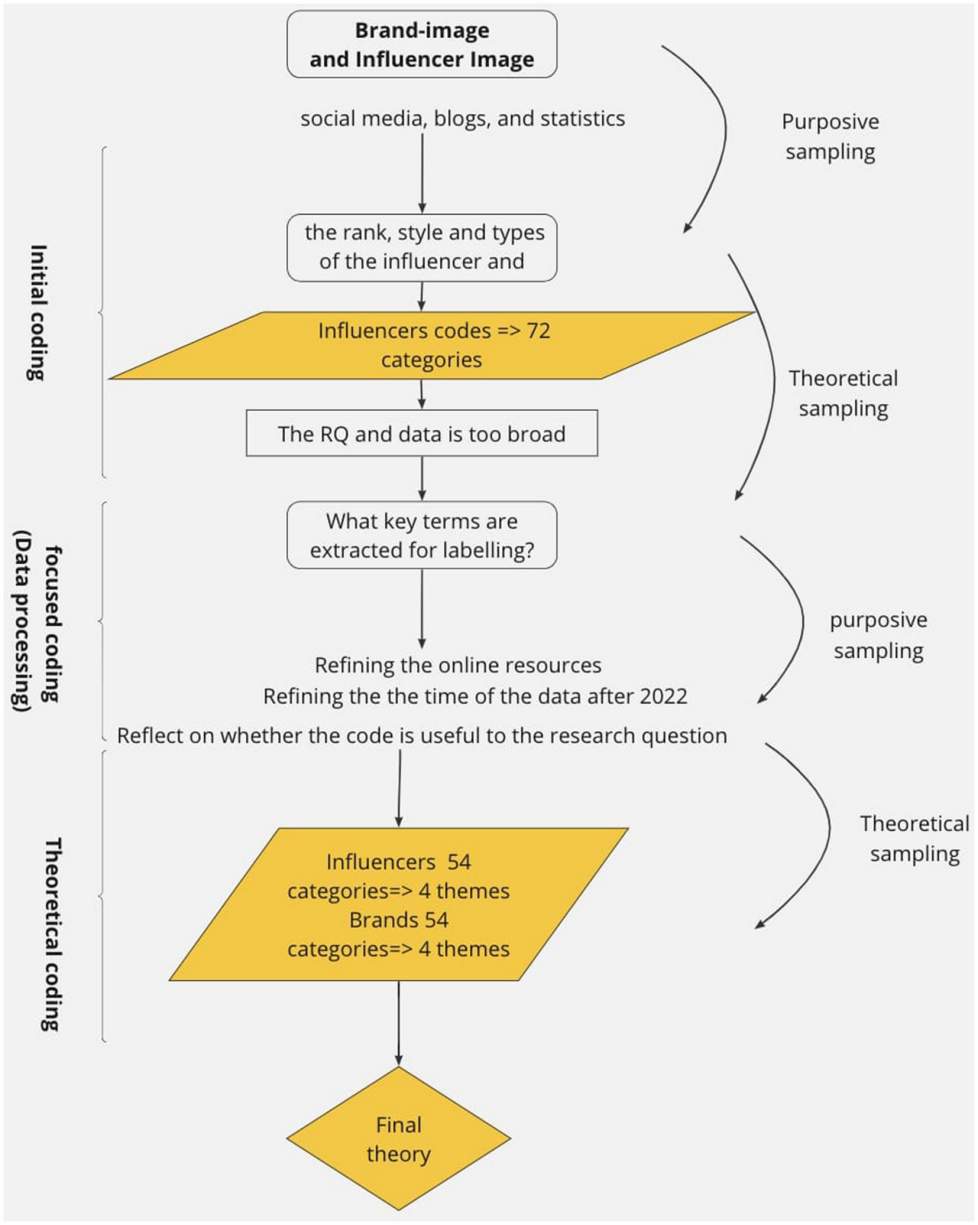
The data collection and coding process.

The coding process segments information into short phrases to label and summarize each piece of data, and the codes are conceptual to reach generalizability (Chametzky, 2016). The organization and development of codes bridge data and theory by defining what the data means. There are mainly three coding styles: Line–by–line, word–by–word, and incident-by-incident. The main coding style we are adopting is the incident-by-incident coding style since a large amount of our data is from empirical statistics, and the data would involve age and ethnicity. Line-by-line and word–by–word coding styles are also adopted when analyzing blogs and social media resources (Brink et al., 2006). The procedure we used purposive sampling and theoretical sampling. “Constant comparative methods” is implemented throughout the coding process (Glaser and Strauss, 1967). The constant comparative method helps the researchers extract code critically, especially in cases where the theory and the codes contradict. According to Charmaz, grounded theory consists of at least two steps: the initial coding stage and the focused coding stage (Kathy, 2006). We have also included the theoretical stage because we have a substantial amount of data to analyze, and the inclusion of the theoretical stage may assist in making the code more meaningful as shown in Figure 2.

#### 4.3.1. Initial coding stage

Charmaz's idea of the initial coding stage mines the early data by segmenting each data piece into words and lines. The openness of initial coding should inspire researchers' thinking (Kathy, 2006). It is the stage where the goal is to narrow down the research questions by finding patterns in the codes. Common questions researchers are what this data suggests, from whose point of view, and what theory could this make of? The initial coding stage encourages researchers to create newly founded data instead of codes from existing categories. In practice, the initial coding stage provides researchers with speed and spontaneity. However, researchers need to be careful about how to fit the code is to the research questions, especially at this stage they need to use “constant comparative methods” to distinguish and analyze the similarities and differences of codes at each analytic work level and stay close to the data (Glaser and Strauss, 1967). The initial coding stage helps us understand how the coding process develops, why and when to appropriately change the process, and the pros and cons of the process (Kathy, 2006).

#### 4.3.2. Focused coding (data processing)

Focused coding refines the data by comparing data and codes generated from the initial coding process and selecting those that repeat and are of importance. It is an emergent process where the researchers have the flexibility to always move back and move forward with the coding process through reflexivity and comparative coding methods (Flick, 2014). For instance, during the focused coding phase, we selected the age of the influencers as a factor that affects “consumers” purchasing behavior. However, we quickly realized that the age of influencers is only one of the minor factors that influence an influencer's influencing style. In contrast, the influencing style is the factor that influences consumers' purchasing decisions. Consequently, age would be a code, while the actual category is “influencing styles”.

#### 4.3.3. Theoretical coding

According to Charmaz, theoretical coding is a more complex process that follows focused coding. Therefore, we decided to include the theoretical stage as an additional step. Glazer created this method to allow researchers to establish the relationship between the codes developed through the focused coding process (Walker and Myrick, 2006). The theoretical coding process is integrative, allowing us to integrate codes from focused coding to our theory to clarify our context, conditions, and theories. It also enhances the sampling process (theoretical sampling). There are 18 coding families to help decide how to code and what to code and 6 of them are constantly being used: Causes, Context, Contingencies, Consequences, Covariances, and Conditions. It is common for researchers to find many codes in the initial coding process and they are sometimes irrelevant to the research topic. So, those 6 code families could be used to identify which codes are relevant. For example, the context of our setting is an E-commerce business, so codes such as the influencers' political views have very little to do with the research questions because influencers' political views do not normally have direct causes to consumers' purchasing behaviors (Kathy, 2006).

#### 4.3.4. Synthesis and reconstruct theory stage

In the final stage of our research, we integrate the theories derived from the literature review and coding. The concepts of actual-self and ideal-self have been previously theorized in the literature. However, Meng proposed the concept of projected-self, which has not been explicitly described by many studies (Meng and Wang, 2019). The codes of brand-self and influencer-self have been derived through a thorough examination of various online resources (Hermanda et al., 2019). Our research methodology aligns with Charmaz's concept of grounded theory, which advocates for utilizing both practical and theoretical resources in developing theories. Integrating these various sources of information has allowed us to produce a comprehensive and well-rounded understanding of the topic under investigation.

## 5. Results

### 5.1. A six-step model of recommendation

This model comprises 6 practical steps to achieve a recommendation system that would fit the new age of AI. The first five steps in the process provide a comprehensive understanding of consumers by considering five distinct aspects. Utilizing this information, the final step is to formulate recommendations based on the insights obtained.

**Figure 3:**
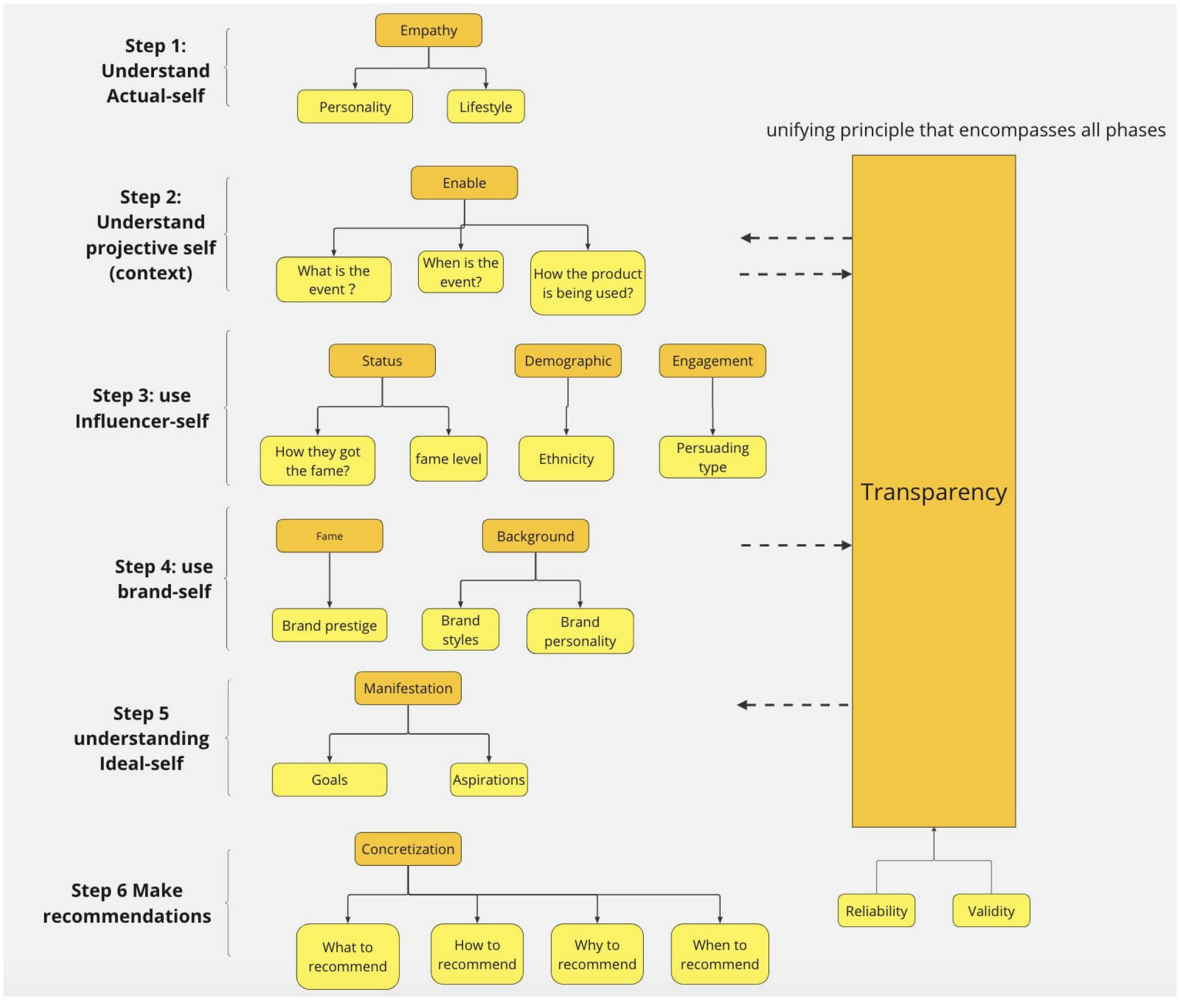
Diagram of metrics for the identities of interest in this study.

Figure 3 highlights a systematic approach to measuring self-concept in consumers and making recommendations. Step one entails determining the actual-self of individuals based on their personality and lifestyle, which can be obtained through their past shopping experiences, social media information, and online surveys. The objective of step two is to unveil the projective-self, exploring why, when, and how consumers utilize the product in question. This process is achieved through conversational interactions with a chatbot, which elicits more personalized information from consumers than a traditional search engine query. Step three involves anchoring the influencer self by carefully attaching influencer information to products and measuring the influencer self-image through factors such as the source of their fame, degree of popularity, ethnicity, and persuading type. Step four introduces the brand self, where brand information is attached to products, and the brand self-image is evaluated based on brand prestige, style, and personality. In step five, the ideal-self is inferred by assessing consumers' purchasing goals and aspirations through a series of questions. Finally, based on the information gathered and analyzed in the previous steps, a scoring system is employed to evaluate the metrics, and recommendations are made to consumers.

#### 5.1.1. Step 1: measure actual-self identity

The initial step in comprehending consumer behavior involves the identification of the consumer's actual-self, or true identity, which encompasses their self-perception and how they wish to be perceived by others. During the literature review phase, it was recognized although academics propose many metrics, few of them could be implemented in the E-commerce settings (Graeff, 1996). Another problem we found was that many actual-self surveys have an excessive amount of metrics. This is impractical when we need to implement those metrics into a recommendation system, at least at this research stage. So, their metrics are significantly summarized by our practice.

Through a thorough literature review, we identified two overarching themes: personality and lifestyle. Our findings indicate that impulsiveness, neuroticism, and innovativeness are the key personalities that influence purchasing behavior. Impulsiveness refers to a consumer's tendency to make rapid, spontaneous decisions without much consideration, which may result in regret and high return rates. Impulsiveness is also one of Graeff's metrics (Youn and Faber, 2000; Verplanken and Sato, 2011). Neuroticism, on the other hand, suggests that consumers may be anxious and seek comfort in their purchasing decisions, leading to less risky and more convenient choices. This term is chosen since it covers meanings such as tense and uncomfortable. Furthermore, innovation-seeking consumers are more inclined to take risks and desire change. This term covers terms such as bold, exciting, non-conformist, and Changeable. Lifestyle also significantly impacts purchasing behavior, with conscientious consumers perceived as organized and likely to make logical, responsible decisions, subscribing to products they regularly need and avoiding regret (Brown and Taylor, 2014). Furthermore, hedonic consumers are drawn to luxury products and seek the finest offerings (Arnold and Reynolds, 2003; Jones et al., 2006). We expect the actual-self identity on consumers have them make loss-prevent decisions such as they would be more prone to by practical products and they are more influenced by influencers that are more similar to their current status.

#### 5.1.2. Step 2: unmasking projective-self identity

Good recommendations need to understand consumers' purchase context such as the occasion, event timing, and product usage. These basic questions reveal the consumer's self-image and purchasing context. One effective approach to comprehending the consumers' context is through conversational interaction, such as ChatGPT. This not only enables consumers to express their needs to the e-commerce platform but also grants them the flexibility to continuously refine and adjust their requirements. However, one of the difficulties in comprehending the consumer's projective self is their potential inability to articulate their preferences. To address this, we propose inquiring about the brands and influencers influencing their decision-making process. This will improve understanding of the consumer's projected self and lay the groundwork for making informed recommendations.

#### 5.1.3. Step 3: anchoring influencer-self images

The concept of influencer-self is a multi-dimensional construct that can be evaluated through three key aspects: influencer status, demographic, and engagement. Influencer status encompasses two dimensions: the source of fame and the level. The source of fame can range from social status, such as in the case of Kate Middleton, to creative work and unique personalities in the entertainment industry, such as Rihanna, Justin Bieber, and Serena Williams. Alternatively, some influencers, such as Cristiano Ronaldo, attain recognition through their expertise and professional accomplishments.

Demographic information on influencers is a complex task that involves various factors such as culture, nationality, history, religion, and language. During the code development, we found normally there are over 10 groups of ethnicity groups. However, this information has been broadly categorized into African descent, Asian descent, and European descent (Agyemang et al., 2005; Lee and Ramakrishnan, 2020; European Ethnic Groups and Nationalities, 2022). The development of this category of codes is to test consumers' reactions to influencers with similar or different ethnical appearances.

Finally, engagement refers to the persuasive styles of influencers, which can be grouped into two broad categories: expressive and observant. Expressive influencers are characterized by their outgoing nature and effective engagement with their followers, while observant influencers are known for carefully analyzing their followers and the brand. In the initial coding stage, we chose influencers with much online information, such as Oprah Winfrey, Barak Obama, and Tim Cook. Initially, we coded them according to their personality types such as introvert and extrovert, their political views such as conservative and liberal. However, those codes do not directly impact their followers in the Ecommerce settings.

Consequently, in the focused coding stage we created the category of persuading styles that involves expressive and observant. The example for observant influencers includes Apple's CEO, who is not very vocal and expressive but has a great impact on people in the technology field as a role model. At the same time, most celebrities such as Oprah Winfrey would be considered as expressive influencer.

#### 5.1.4. Step 4: introducing brand-self images

Understanding consumer behavior and marketing strategies involve an assessment of brand fame and background. The fame of a brand is categorized into three groups: luxury, premium, and mass-market. Armani and Hermes, for example, are associated with high prestige, exclusivity, and strong brand heritage (Cuomo et al., 2019; Lee, 2021). These brands often have associated with high-status individuals and are known for their exceptional quality, attention to detail, and craftsmanship. Premium brands like Tommy Hilfiger and Ralph Lauren distinguish themselves through design and quality but invest less in brand storytelling. Mass-market brands, such as Zara and Uniqlo, are considered the least prestigious and target a wider audience with products that prioritize practical attributes such as quality, price, and functionality (Bhat and Reddy, 1998).

The background of a brand is further divided into two categories: brand style and personality. Brands often possess unique styles that establish their market niche, such as classic brands like Burberry and Givenchy or trendy brands like H&M and Zara, which follow current fashion designs and target a young audience easily influenced by the latest trends. Streetwear brands, such as Supreme, produce casual and fashionable clothing inspired by urban and street culture and target a young audience seeking to be perceived as cool and unique (Tungate, 2005). During the coding process, we found that some brands, such as Luis Viton, can have several brand styles. The brand personality theory was developed to analyze the psychological distance between brands and consumers. Brands possess personalities much like individuals and can be regarded as modest or demanding, decent or eccentric, tough or sensual (Heine et al., 2018). Those brand personality categories were coded because they contain several codes. For example, touch or sensual also contains the code feminine or masculine and hard or soft. Modest or demanding also contains dominating or submissive and adventurous or timid. Those codes of influencers and brands could be utilized to portray a consumers' projected self. In other words, it is a mean to quantify consumers' self identities of actual or projective states under a particular context by using codes that describe the brands and influencers. Therefore, the codes could be extensive.

#### 5.1.5. Step 5: inferencing ideal-self

The concept of the ideal self, also known as the desired self or idealized image, represents how individuals would like to perceive themselves. It operates from a perspective of growth and advancement and is centered on enhancing one's self-concept (Toyama, 2022). Regarding e-commerce, the goals of interest are consumers' purchasing goals, which can be influenced by the brands and influencers they admire. When individuals are operating from their ideal self, they are more likely to engage in spending behaviors aimed at enhancing their self-image rather than maintaining their current status quo. The ideal-self could be attained through asking consumers questions that would be inspiring to see what person they would like to be after purchasing and using the products. Those questions could be asked during the chatting process. The ideal-self plays an important role on how the projected-self is formed by operating from what consumers' want rather than need. In other words, their purchasing decisions is influenced by how they want to be in the near or far future. When consumers' ideal-self is more framed in the purchasing context, they intend to have more hedonic behavior such as seek for luxurious products (Liu et al., 2012).

#### 5.1.6. Step 6: make recommendations

The recommendation process consists of all five dimensions of self-concept and provides a well-rounded and balanced recommendation to the consumer. This is achieved through the proper categorization, tracking, and measuring of each aspect of the consumer's self-concept. This information can then be used to train the recommendation algorithm and continually improve its understanding of the individual consumer, enabling it to make increasingly personalized recommendations in the future. By considering all the important parts of the consumer's self-concept, the recommendation system can fully understand the consumer's preferences, needs, and motivations. This helps the system make more accurate and useful suggestions.

### 5.2. Experimental procedures

In total of 500 participants from different ethnical backgrounds would be recruited from channels such as university and MTurk. A lottery is set up as an incentive for the participants who attend the experiments from the university. The primary instrumental tool to gather data is a fake E-commerce website linked to a controllable SQL database. The website is built using React framework linked to a fire store database to ensure maximum flexibility over the data collection process. Users' clickstream data is collected through a scoring system that measures against the metrics for influencer and brand self-image. This process is automated using JavaScript code. After that, the data is sent to a machine-learning model. The model would analyze data and predict consumer behavior.

**Figure 4:**
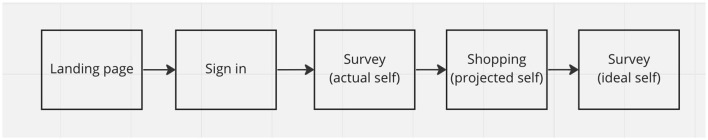
Website flow.

**Figure 5:**
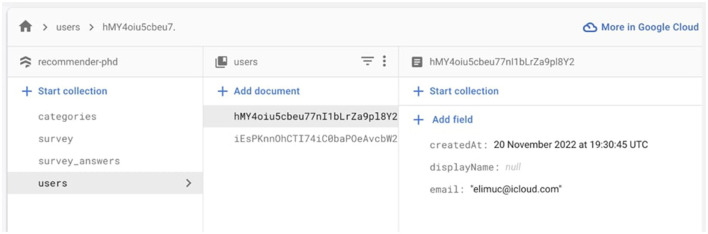
Database of the users.

As the flow chart illustrated above in Figure 4, the website works in a one-way flow. There are five parts to it: It starts with instructions on navigating the website. Then the user needs to sign up/sign in to continue. The sign-in function ensures that the system can recognize the user and record the user's behavior in the database, such as time, unique token, and choices, as shown in Figure 5. The sign-in process also helps the users to agree with the terms and privacy policy. After signing up, the system would recognize which user is taking the experiment and send this user to a survey page. The questions on this page are formed with Likert scales. It takes the basic demographic information of the users as well as a survey to test their actual self-image. Once the users make their choices, a score gets sent back to the backend database to trigger the next step, simulating their shopping experience on an E-commerce shopping page. On this page, users are instructed to only choose one product under a given context instructed. In order to make sure the user reads the information about the product carefully, they will have to click on a new page that offers detailed brand information and influencer information. Once they choose, the data, such as time and all the tags linked to this particular product, get automatically calculated and sent back to the database for further analysis. The final questionnaire is then followed to test the user's ideal self.

**Figure 6:**
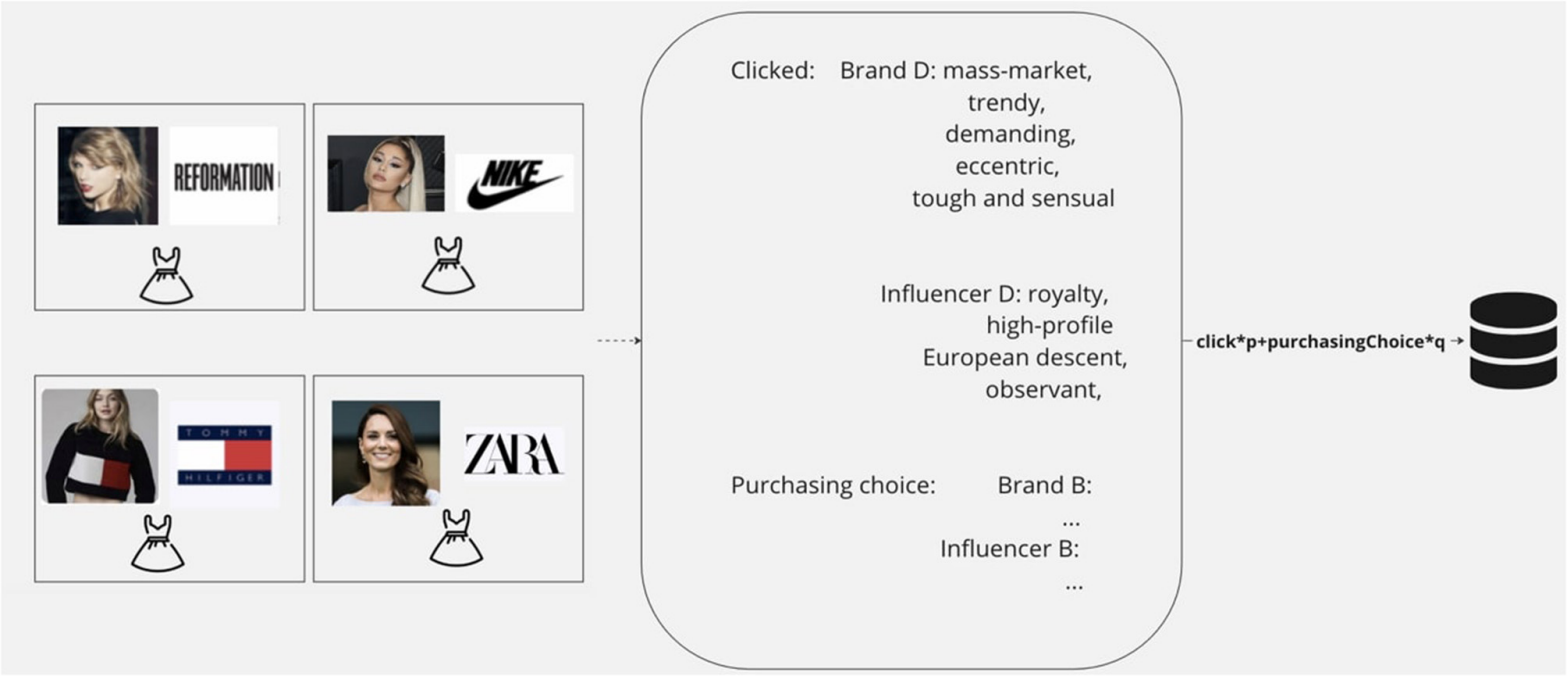
The scoring system.

The influencer self and brand self are measured by a series of metrics using a scoring system, as shown in Figure 6. The metrics to measure influencer self-image are as follows: fame source, fame level, ethnicity, and persuading type. The metrics for a brand's self-image include the brand's prestige, style and personality. The projective self is measured by using a scoring system as shown in Figure 6: projective self = click score^*^p + purchasing choice score^*^q. Based on the user's clicking behavior and purchasing behavior, a total score for each metric was calculated. The actual self and ideal self are measured using the metrics of influencer self and brand self in the form of a survey. The metrics are chosen from various previous literature, such as Belk's brand personality framework. The metrics are kept minimal to ensure the lightweight processing of the website and data analysis software under the principle that the metrics are sufficient to represent the shopping experience.

### 5.3. Control of experiments

Our method is effective since it is built upon currently successful recommendation systems. The shopping experience simulates a standard E-commerce website where the user starts with the intention of what they are looking for (context) and then adds the product to the shopping cart. Furthermore, the self-construal theory has been studied extensively for over 40 years. The evidence from many pieces of research could be compared with our study. Any product could be chosen as long as they have a sense of brand identification and influencer identification. This experiment makes use of fashion products such as clothing. The bias of this experiment is carefully controlled to consider the balance of ethnicity, gender, and social classes. For ethnicity balance, the influencers are from various ethnic backgrounds; for gender balance, the Products are chosen to interest males and females. The products do not show the pricing information to eliminate the bias of participants' financial status. This ensures that users make choices based on their preferences but not financial restraints. The data collection process is controlled to ensure the least errors occur. First, we designed the website to be one-way flow and easy to navigate so that the users are more likely to choose according to the instruction. Second, users might choose a product according to the functionality and aesthetics of the product instead of the brand or the influencer associated. Therefore, the product selected in the experiment is abstract in their functionalities and aesthetics. Finally, to motivate the consumers to choose with caution, we provide a minimal number of products that each present the metrics we want to measure. Furthermore, incentives in the instruction, such as “We believe you can find the item that is most suitable for you”. To make sure again that they selected the product with caution, we double-check how long they have spent in the choice-making process and what products they have clicked. A pertest of 100 people is conducted to ensure that the brands and influencers are recognized and diversified and could be recognized by the participants. It also tests how diversified our participants are. After making sure the set-up of the experiment is valid, the data would be collected on a small scale. The data collected from the experiment will be checked against Belk's brand personality framework (Belk, 2014a) to improve the conceptual model to improve the experiment further. The demographic data will be analyzed to decide whether or not to change the channel of recruiting participants forthcoming experiment. For example, in Mturk, they offer better control of participants representing various demographics.

### 5.4. Unifying principle that encompasses all phases

The fundamental principles that underlie all phases of the recommendation process are the transparency of consumers' preferences, needs and motivations. For a recommendation system to be effective, it must possess a thorough grasp of its customers, including their context, history, preferences, requirements, and motivations. This comprehension is essential for the precision of recommendations, but it must also be adaptive and capable of continuously growing in response to newly accessible data. To do this, the recommendation system must be flexible and transparent, allowing for continuous evaluation and improvement of its algorithms, data sources, and decision-making processes. This ensures that recommendations remain relevant and practical, even as consumers' needs and behavior evolve (Jesse and Jannach, 2021).

## 6. Conclusion

### 6.1. Summary

The study applied grounded theory and literary synthesis to solve two issues: As a first issue, recommendation systems suffer from a dearth of psychological frameworks that serve as its foundational principles. Second, the technology behind recommendation algorithms is lacking in terms of actual metrics. Several codes that were not implicit in the current literatures in the field of E-commerce settings were retrieved using grounded theory, and the application of synthesis of literary analysis ensures that the summary theory is exhaustive.

### 6.2. Ethical concerns

The Loughborough University Ethics Committee has accepted the research based on stringent criteria. Examples of what the committee looks for include whether the study involves international collaborations with a high level of ethical risk and if you are collecting identifiable personal information or sensitive personal information, what is the legal basis for processing 'personal information' for your study. The planned empirical research technique and the questionnaire sample that will be employed have also been ethically approved.

Although the research has passed ethical approval by Loughborough University, there are potential ethical issues for recommendation systems in general. There are two main ethical concerns: privacy and system bias. On a single platform-level, the privacy issue is crucial because the algorithm will understand users' deep psychological needs and states. It is essential for retailers to not only protect consumers' personal data from malicious hackers but also make sure the recommendation system cannot identify each consumer (Ricci et al., 2015; Kumar and Thakur, 2018). In a cross-platform recommendation system, where the future of recommendation systems is going to be, privacy is a problem because not only does each company need to decide how to keep their data private, but they also need to decide how and what to share with other platforms. The solution has been researched by authors such as Qi et al. (2018) and Cui et al. (2021). The common approaches to achieve this goal are pseudonymization and anonymization of consumers' data (the formal approach is where companies encrypt consumers' personal information in their database, and the latter approach is where the data collection was conducted in a way that the consumer is not identifiable) (Hintze and El Emam, 2016). Privacy is also the responsibility of the law enforcement departments of each country to protect consumers' rights. Currently, GDPR is considered "the toughest privacy and security law in the world (Ben, 2020).

The second concern is the fairness of recommendation systems. It is crucial because the algorithm could impact the consumers' decision-making process, which is one of the reasons why the transparency of algorithms is crucial. In other words, should it be customers' right to demand their interactions with trusted recommendations instead of being assigned to one whose behavior is not obvious (Paraschakis, 2017)? Our research aims to solve this problem by making the recommendation system more explainable using psychological metrics, which would help consumers make better decisions about whether or not to trust a specific recommendation system.

### 6.3. Theoretical implications and practical implications

Despite the extensive research conducted over several decades exploring various aspects of self-concepts, our study represents a unique contribution by synthesizing the existing theories and applying them specifically to the context of E-commerce. In doing so, we have taken into consideration the practicality, necessity, and validity of the theory, intending to further develop and test the theory through empirical studies in the future. Our study represents an important step toward applying self-concepts in E-commerce settings. One of the problems that the coding process solves is that there is lack of studies done in particular areas of our research questions. For example, the coding process derived influencer persuasive styles as expressive and observant, whereas very little study has identified this due to the niche scenario we are targeting. Collins only described those two types in his research (Collins, 2006). By combining coding with our research, we can generate a comprehensive theory. Furthermore, the five self-identities use different metrics, which could be a potential problem when analyzing the results, according to Waugh (2001). On the other hand, using different metrics provides us more dimensions for each self-identity.

Large companies such as Amazon are researching in the direction of neurosymbolic AI and neuro-symbolic AI. In other words, these systems are bridging the gap between computers' logic, such as mathematics, and humans' logic, which is highly influenced by culture and societal structures (Mao et al., 2019; Garcez and Lamb, 2020; Liang et al., 2020; Larry, 2021). Our research can help those big companies design the human logic behind their models. The principle of this research endorses the philosophy of viewing the recommendation system as a model with psychological reasoning embedded. The advantage that those big companies have is that they have rich data to analyze the psychological reasoning of consumers. Another advantage is that they have abundant computation power, so the model would run faster and be more accurate.

Alternatively, the algorithm we propose offers small organizations and entities opportunities to develop their own algorithms, as evidenced by discussions between the researcher and multiple e-commerce website owners, including a rapidly growing cosmetics company in the UK. These smaller entities often lack the resources and capacity to conduct extensive research and develop customized recommendation systems. The researcher also discussed the feasibility of our proposed model with several experienced programmers in the industry, and it is believed that the model presents a viable solution that a wide range of e-commerce settings can easily adopt.

### 6.4. Limitations and research agenda

We anticipate conducting empirical studies to accomplish the following research objectives in the future. Firstly, we hope to clarify the relationship between the five self-concepts employing illustration. Secondly, despite its simplicity, our new algorithm is expected to make constructive predictions. These predictions are expected to support the theory presented in this paper. Lastly, we plan to investigate and discuss the limitations of current state-of-the-art algorithms, such as the cold-start and data sparsity problems, under the new study instrument. We believe this recommendation system algorithm will be more explainable. It is anchored in a psychological framework that provides a measurable metric for the current machine learning (artificial intelligence) algorithms. Ultimately, we hope to gain more precise insights from real-time data to better understand the causes of the cold start and data sparsity problem.

The effective method is built upon a simulated E-commerce website to testify participants' in-time and real reactions to their purchasing experience. The shopping experience simulates a standard E-commerce website where the user starts with the intention of what they are looking for (context) and then adds the product to the shopping cart. The website is built according to the six-step model. In the analysis process, the gaps between the 5 self-concepts and the accuracy of the recommendation will be measured to explore the possibility of further improving the algorithm.

The limitations of this study are primarily rooted in its reliance on grounded theory methodology, where the validity of the codes generated is contingent upon the researcher's interpretation (Cutcliffe, 2000). The empirical testing of the theory proposed in this study could help address these limitations. Additionally, the study is also limited by the unstructured nature of the online data sources, which presents challenges in processing and interpreting the data. This required the researcher to employ critical thinking and careful coding to extract relevant information. Despite these limitations, this study offers valuable insights into the relationship between self-concepts and optimizing personalized recommendations in e-commerce. Further research, utilizing structured data sources as suggested before, it is necessary to further validate and expand upon the findings of this study.

The research agenda consists of four sub-areas that are connected with this paper: First, over the e-commerce context, the application scope of this proposed method can be expanded to other studies of digital behaviors and the service economy via digital interfaces where customer's construal-self cannot be directly observed but implied. Second, it awaits to determine how the proposed recommendation system could be utilized in the era of rapidly evolving artificial intelligence in terms of the algorithm's resilience and transferability. Thirdly, it is on our agenda to discuss how practical, affordable, and easy-to-implement our strategy is in terms of the cost of key technologies and the method by which this system collects data from users. Since there are pros and cons associated with each data mining process in terms of how long they take, how much computational power they require, and which data structure they require, we aim to identify the most efficient method for analyzing and mining the data we collected from the gaps of 5 self concepts. Last, alongside the clickstream data this paper intends to collect and adopt for consumer identities, the adoption of biological response collector such as eye tracker, galvanic skin response or electroencephalogram tools, will enrich the findings from consumer's browsing contents and choices for a large-scale machine learning.

## Data availability statement

The original contributions presented in the study are included in the article/supplementary material, further inquiries can be directed to the corresponding author.

## Author contributions

In this study, YF studied the application of the construal-self theory to the algorithms of existing recommendation systems. YF has studied the approach's practicability, necessity, and validity, aiming to develop and test it through empirical research in E-commerce environments. The coding approach from grounded theory author applied tackles the paucity of studies in some areas of our study problems. Furthermore, the research also discussed the practicality of employing this method in the market notably for small E-commerce firms since they frequently lack the means and capacity to conduct extensive research and development of customized recommendation systems.
